# A Traumatic Dislocation of the Atlas from the Axis in a Patient with Atlantooccipital Assimilation

**DOI:** 10.7759/cureus.4402

**Published:** 2019-04-06

**Authors:** Raja Gnanadev, Basem Ishak, Joe Iwanaga, Marios Loukas, R. Shane Tubbs

**Affiliations:** 1 Miscellaneous, Seattle Science Foundation, Seattle, USA; 2 Neurosurgery, Seattle Science Foundation, Seattle, USA; 3 Medical Education and Simulation, Seattle Science Foundation, Seattle, USA; 4 Medical Education and Simulation, St. George's University School of Medicine, St. George, GRD

**Keywords:** trauma, craniocervical, congenital, assimilation

## Abstract

Atlantooccipital assimilation is defined as the complete or partial fusion of the caudal portion of the occiput with the cranial portion of the atlas. We report an extremely rare case of a traumatic dislocation of the atlas from the axis (C1 from C2), which lead to internal decapitation in a patient with atlantooccipital assimilation. This report reviews the background of atlantooccipital assimilations and their clinical relevance.

## Introduction

Atlantooccipital (AO) assimilation is defined as the complete or partial fusion of the occiput with the atlas [1]. The prevalence of atlantooccipital assimilation ranges from 0.08%­-3% and may be congenital or acquired [2].

The dislocation of the atlantoaxial joint often leads to internal decapitation. The primary mechanism of injury is trauma: dives, falls, assault, and motor vehicle accidents [3]. We report an extremely rare case of traumatic dislocation of the atlas from the axis, leading to internal decapitation in a pediatric patient with AO assimilation. This report reviews the background and clinical relevance of AO assimilations.

A review of the literature was conducted through Google Scholar, PubMed, and Google Books using keywords including, but not limited to, “atlantooccipital assimilation,” “atlantooccipital fusion,” “atlantoaxial dissociation,” “atlantoaxial dislocation,” and “basilar invagination.” This search yielded no literature involving AO assimilation and a C1-C2 dislocation with internal decapitation.

## Case presentation

A 16-year-old male presented to the emergency department following a high-speed front-end collision with another vehicle. He was restrained and occupied the front seat of the vehicle that was struck head-on. The speed at the time of the collision was estimated to be in excess of 79 miles per hour. On arrival, the patient was unconscious and not breathing independently. Attempted intubation at the scene was unsuccessful and the patient was pronounced dead soon after arrival to the hospital.

**Figure 1:**
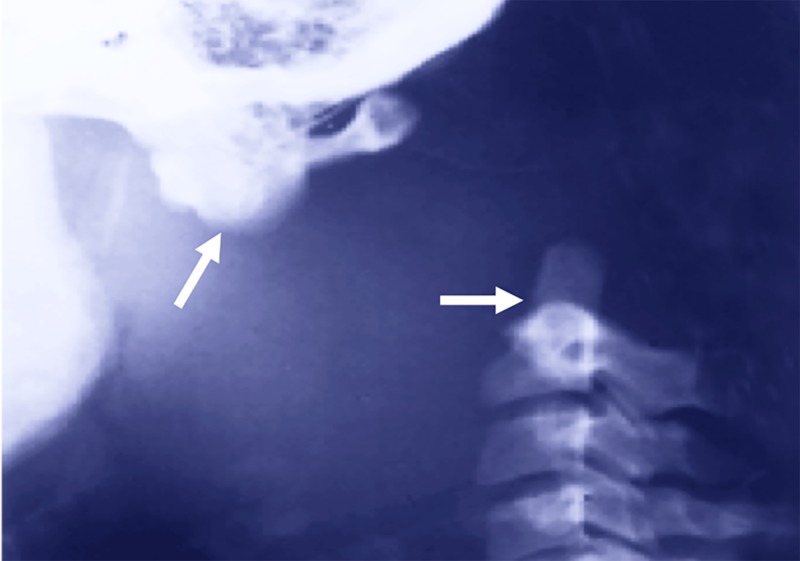
Lateral radiograph of the craniocervical junction and upper cervical spine noting that the white arrows point to the assimilated atlas (left) and bare odontoid process of C2 (right)

Radiographs at the time of the incident noted internal decapitation with AO assimilation specifically between the anterior arch of the atlas and the basiocciput (Figure 1). No fractures were noted, including the odontoid process. There was no known past medical or surgical history and the AO assimilation was unknown to the family.

## Discussion

Two pathologies were observed in the present case: AO assimilation and complete dislocation of the atlantoaxial joint with an intact odontoid process. AO assimilation is a common anomaly to the upper cervical region [2,4]. The prevalence has been cited as 0.08%-3.0% of the population, but more recent studies cite an incidence near 1% [5-6]. Typically an incidental finding, AO assimilation is due to ponticles from the anterior or posterior arch of the atlas [2,7].

For congenital AO assimilation, the embryology has been described as a failure of differentiation between somites. Specifically, the caudal base of the occiput is derived from the fourth somite and the cranial portion of the atlas from the fifth somite [2]. Abnormalities in the odontoid process, including congenital hypoplasia, have been associated with AO assimilation [2,8]. AO assimilation may be acquired as a result of post-traumatic deformities, infections, inflammatory conditions, or tumors near the craniocervical junction [5-6].

Clinically, the initial visualization of AO assimilation is with X-ray, followed by multiplane, multi-echo magnetic resonance imaging (MRI) and computed tomography (CT) with three-dimensional (3D) reconstruction [1]. As mentioned, AO assimilation is typically entirely asymptomatic. The literature describes initial symptoms that are apparent only in the second through fourth decades of life, particularly those related to the regional spinal cord or vascular compression. Symptoms range from transient neck pain and headaches to paresthesias and weakness of the upper extremities [7,1].

The neurological symptoms associated with AO assimilation are likely related to basilar invagination [2,8]. Although it is clear that the two are distinctly related, the literature remains inconsistent with AO assimilation causing basilar invagination or vice versa. Part of this discrepancy may be due to the authors’ failure to distinguish basilar invaginations (congenital) from basilar impressions (acquired) [1,8]. The neurological symptoms associated with AO assimilation are considered directly related to the cranial portion of the dens crossing the plane of the foramen magnum, McRae’s line [7]. The literature is notable for sudden death in a 24-year-old patient with AO assimilation where the foramen magnum had a nearly 50% reduction in anteroposterior dimensions [9].

AO assimilation and acquired Chiari I malformation have also been reported in the literature [10]. Though the pathophysiologic mechanism is different, both AO assimilation and Chiari I malformation neurologic symptoms are due to neural tissue compression at the foramen magnum. In a Chiari I malformation, herniating cerebellar tonsils compress neural tissue at the foramen magnum. Both presented with dyspnea and central sleep apnea [11-12].

Trauma as the sole etiology of atlantoaxial dissociations is rare, and in our case, it is associated with a congenital malformation at the atlantooccipital joint. The etiology is often multifactorial, but individual causes include congenital relationships and inflammatory etiologies. Congenital associations include Down syndrome, Goldenhar syndrome, and Morquio syndrome. An incidence of 23%-83% of atlantoaxial involvement is also reported in adults with rheumatoid arthritis, predisposing them to atlantoaxial dissociations [4].

Atlantoaxial dislocation is defined as a distance from the atlas’ anterior arch to the odontoid process greater than 3 mm in adults and 5 mm in children. A distance greater than 7 mm is likely to require surgical fusion [4]. Internal decapitation, in this case, would likely tear ligaments typically connecting the axis and dens to cranial structures. It is, therefore, important to discuss the regional ligaments and structures expected to be torn.

Along the spinal cord, the posterior longitudinal ligament and the ligamentous articular capsule of the atlantoaxial joints would tear anteriorly, the tectorial membrane posteriorly, and the transverse ligament laterally. The ligaments attaching the dens locally would also tear the apical ligament of the dens, alar ligaments, superior and inferior longitudinal bands of the cruciform ligament, and the accessory atlantoaxial ligament [13].

As illustrated by our case, good knowledge of the variations of the craniocervical junction can better help in interpreting imaging in trauma patients [14-20].

## Conclusions

Interpreting post-traumatic imaging necessitates a good working knowledge of the more common anatomical variations in the region of the body being imaged. AO assimilation should be assessed when viewing imaging of the neck and craniocervical junction.

## References

[1] Electricwala AJ, Harsule A, Chavan V, Electricwala JT (2018). Complete atlantooccipital assimilation with basilar invagination and atlantoaxial subluxation treated non-surgically: a case report. Cureus.

[2] Standring S (2016). Gray’s Anatomy: The Anatomical Basis of Clinical Practice.

[3] Yoganandan N, Pintar F, Baisden J, Gennarelli T, Maiman D (2004). Injury biomechanics of C2 dens fractures. Ann Proceed Assoc Adv Automot Med.

[4] Yang SY, Boniello AJ, Poorman CE, Chang AL, Wang S, Passias PG (2014). A review of the diagnosis and treatment of atlantoaxial dislocations. Global Spine J.

[5] Iwata A, Murata M, Nukina N, Kanazawa I (1998). Foramen magnum syndrome caused by atlanto-occipital assimilation. J Neurol Sci.

[6] Sharma DK, Sharma D, Sharma V (2017). Atlantooccipital fusion: prevalence and its developmental and clinical correlation. J Clin Diagn Res.

[7] Kulkarni JV, Kulkarni R (2003). Atlanto-occipital fusion - report of two cases. J Anat Soc India.

[8] Smith JS, Shaffrey CI, Abel MF, Menezes AH (2010). Basilar invagination. Neurosurgery.

[9] Vakili ST, Aguilar JC, Muller J (1985). Sudden unexpected death associated with atlanto-occipital fusion. Am J Forensic Med Pathol.

[10] Kimura Y, Seichi A, Gomi A, Kojima M, Inoue H, Kimura A (2012). Acquired Chiari malformation secondary to atlantoaxial vertical subluxation in a patient with rheumatoid arthritis combined with atlanto-occipital assimilation. Neurol Med Chir.

[11] Tubbs RS, Chern JJ, Muhleman M, Loukas M, Shoja MM, Oakes WJ (2013). Lateral compression of the foramen magnum with the Chiari I malformation: case illustrations. Child’s Nerv Syst.

[12] Tubbs RS, Lancaster JR, Mortazavi MM, Shoja MM, Chern JJ, Loukas M, Cohen-Gadol AA (2011). Morphometry of the outlet of the foramen magnum in crania with atlantooccipital fusion. J Neurosurg Spine.

[13] Tubbs RS, Salter EG, Oakes WJ (2004). The accessory atlantoaxial ligament. Neurosurg.

[14] Shoja MM, Ramdhan R, Jensen CJ, Chern JJ, Oakes WJ, Tubbs RS (2018). Embryology of the craniocervical junction and posterior cranial fossa, part I: development of the upper vertebrae and skull. Clin Anat.

[15] Shoja MM, Ramdhan R, Jensen CJ, Chern JJ, Oakes WJ, Tubbs RS (2018). Embryology of the craniocervical junction and posterior cranial fossa, part II: embryogenesis of the hindbrain. Clin Anat.

[16] Shoja MM, Johal J, Oakes WJ, Tubbs RS (2018). Embryology and pathophysiology of the Chiari I and II malformations: a comprehensive review. Clin Anat.

[17] Yoon SY, Moon HI, Lee SC, Eun NL, Kim YW (2018). Association between cervical lordotic curvature and cervical muscle cross-sectional area in patients with loss of cervical lordosis. Clin Anat.

[18] Badshah M, Soames R, Ibrahim M, Khan MJ, Khan A (2017). Surface anatomy of major anatomical landmarks of the neck in an adult population. Clin Anat.

[19] Sanchis-Gimeno JA, Perez-Bermejo M, Rios L, Llido S, Bastir M, Blanco-Perez E, Mata-Escolano F (2017). Analysis of the relationship between the double transverse foramen and the possibility of developing clinical symptoms after whiplash. Clin Anat.

[20] Shen XH, Xue HD, Chen Y, Wang M, Mirjalili SA, Zhang ZH, Ma C (2017). A reassessment of cervical surface anatomy via CT scan in an adult population. Clin Anat.

